# Short-term physiological hypoxia potentiates the therapeutic function of mesenchymal stem cells

**DOI:** 10.1186/s13287-018-1007-x

**Published:** 2018-10-11

**Authors:** Ben Antebi, Luis A Rodriguez, Kerfoot P Walker, Amber M Asher, Robin M Kamucheka, Lucero Alvarado, Arezoo Mohammadipoor, Leopoldo C Cancio

**Affiliations:** 1 United States Army Institute of Surgical Research, San Antonio, TX USA; 2 Oak Ridge Institute for Science and Education, Oak Ridge, TN USA

**Keywords:** Mesenchymal stem cells (MSCs), Hypoxia, Normoxia, Bone marrow, Immunomodulation

## Abstract

**Background:**

In the bone marrow, MSCs reside in a hypoxic milieu (1–5% O_2_) that is thought to preserve their multipotent state. Typically, in vitro expansion of MSCs is performed under normoxia (~ 21% O_2_), a process that has been shown to impair their function. Here, we evaluated the characteristics and function of MSCs cultured under hypoxia and hypothesized that, when compared to normoxia, dedicated hypoxia will augment the functional characteristics of MSCs.

**Methods:**

Human and porcine bone marrow MSCs were obtained from fresh mononuclear cells. The first study evaluated MSC function following both long-term (10 days) and short-term (48 h) hypoxia (1% O_2_) culture. In our second study, we evaluated the functional characteristics of MSC cultured under short-term 2% and 5% hypoxia. MSCs were evaluated for their metabolic activity, proliferation, viability, clonogenicity, gene expression, and secretory capacity.

**Results:**

In long-term culture, common MSC surface marker expression (CD44 and CD105) dropped under hypoxia. Additionally, in long-term culture, MSCs proliferated significantly slower and provided lower yields under hypoxia. Conversely, in short-term culture, MSCs proliferated significantly faster under hypoxia. In both long-term and short-term cultures, MSC metabolic activity was significantly higher under hypoxia. Furthermore, MSCs cultured under hypoxia had upregulated expression of VEGF with concomitant downregulation of HMGB1 and the apoptotic genes BCL-2 and CASP3. Finally, in both hypoxia cultures, the pro-inflammatory cytokine, IL-8, was suppressed, while levels of the anti-inflammatories, IL-1ra and GM-CSF, were elevated in short-term hypoxia only.

**Conclusions:**

In this study, we demonstrate that hypoxia augments the therapeutic characteristics of both porcine and human MSCs. Yet, short-term 2% hypoxia offers the greatest benefit overall, exemplified by the increase in proliferation, self-renewing capacity, and modulation of key genes and the inflammatory milieu as compared to normoxia. These data are important for generating robust MSCs with augmented function for clinical applications.

## Background

Mesenchymal stem cells (MSCs) have emerged as potent therapeutic tools for treating lung conditions [1–4], exemplified by their use in numerous small [5–16] and large [17, 18] animal studies as well as early phase clinical trials [19–22] for the treatment of acute lung injury. In the bone marrow, MSCs reside in low oxygen tension (hypoxia), which has been shown to range from 1.5 to 4.2% oxygen depending on the location in the marrow space [23]. Hence, culturing MSCs under hypoxia in vitro can mimic the physiological niche that occurs in vivo and potentially facilitate the retention of the stem cell state. Another rationale for hypoxia culture is to prepare or pre-condition the cells to a similar oxygen concentration they will be exposed to when administered into injured tissue, which has been shown to be in the range of 0.4–2.3% [24]. Since the majority of MSCs do not survive the hypoxia milieu within injured tissue, a pre-conditioning period may permit adaptation prior to their exogenous administration. Typically, however, MSCs are expanded under atmospheric (20.9%) oxygen tension (i.e., normoxia), a process that has been shown to impair their proliferative and differentiation capacity [25, 26] and does not properly recapitulate or prepare them for the in vivo environment.

Clinical applications involving systemic administration of MSCs require a large number of cells (1–10 × 10^6^ cells/kg) to demonstrate a therapeutic efficacy [27–29]. Generating large number of cells necessitates a large-scale expansion in vitro. Yet, due to cell senescence and spontaneous differentiation, MSC expansion in vitro is limited before their therapeutic efficacy is severely diminished. The optimal conditions for generating large doses of high-quality MSCs have been under intense research during the last decade. Various parameters, such as appropriate media, supplement, seeding density, and technologies for their large-scale expansion (e.g., bioreactors) are under constant development to deliver the ideal MSC product.

One such parameter is expansion of MSCs under hypoxia. A large number of studies demonstrated that MSCs cultured in hypoxia exhibit higher rates of proliferation and better retain their stem cell properties [25, 30–33]. Yet, some studies reported conflicting results showing negative or no effects on MSCs by hypoxia [34–36]. Thus, the question whether hypoxia preconditioning should become the standard of care for clinical application of MSCs remains to be answered [37, 38]. Specifically, important questions that require further investigation are the precise oxygen concentration and appropriate incubation time that will provide the greatest benefit in terms of the therapeutic efficacy of MSCs. In this study we attempted to answer these two questions by evaluating the functional characteristics of human and porcine bone marrow MSCs cultured for both long-term (10 days) and short-term (48 h) in hypoxia in comparison to normoxia. Next, once we identified that short-term hypoxia is more suitable, we examined the same functional characteristics under two additional oxygen tensions (i.e., 2% and 5%). The objective of this study was threefold: first, to elucidate whether hypoxia is preferred over normoxia for MSC culture; second, to identify the ideal hypoxia conditions (i.e., duration and oxygen tension) that will provide MSCs with robust therapeutic potency; and third, to precondition the MSCs in preparation for the hypoxia environment they will experience in vivo in hopes of prolonging their survivability and augmenting their function.

## Methods

### Isolation and culture of mesenchymal stem cells

Porcine bone marrow MSCs (pMSCs) were isolated from mononuclear cells (MNCs) obtained from bone marrow aspirates, as previously described [39]. Human bone marrow MSCs (hMSCs) were isolated from MNCs purchased from AllCells LLC (Emeryville, CA, USA). MNCs were cultured on standard culture flasks at a density of 3 × 10^5^ cells/cm^2^ in minimum essential media alpha (a-MEM), supplemented with 15% heat-inactivated, lot-selected fetal bovine serum (Atlanta Biologicals, Atlanta, GA, USA), 2 mM L-glutamine, and 1% antibiotic-antimycotic, regarded as complete culture media (CCM). The media were changed every other day and the cells were allowed to grow for approximately 2 weeks. Once colonies formed, but before they overlapped, the plastic-adherent cells were enzymatically detached using 0.25% trypsin-EDTA and regarded as passage 0 (P0) MSCs.

For normoxia, MSCs were cultured in standard cell culture incubators (5% CO_2_/95% air; 37 °C). For hypoxia, MSCs were cultured at either 1%, 2%, or 5% O_2_ (5% CO_2_/remaining N_2_; 37 °C) using a dedicated hypoxia station (HypOxystation H35, HypOxygen, Frederick, MD, USA). For all assays, P2-P3 MSCs were used and all experiments were performed in triplicates. Unless otherwise stated, all reagents were purchased from Thermo Fisher Scientific (Waltham, MA, USA).

### Flow cytometry

Cells were examined by flow cytometry for the expression of common MSC markers. Human MSC Analysis Kit (BD, Biosciences, San Jose, CA, USA) was used for assessing hMSCs. Cells were stained with pre-conjugated antibodies based on the manufacturer’s instruction. Briefly, hMSCs were incubated with staining buffer containing 1% bovine serum albumin and Fc blocker (BioLegend, San Diego, CA, USA) for 10 min at a cell concentration of 1 × 10^6^/ml in order to reduce nonspecific binding. Then, the antibody cocktail for MSC-positive markers (CD90-FITC, CD105-PerCP-Cy5.5, CD73-APC), and negative markers (CD45-PE, CD34-PE, CD11b-PE, CD19-PE, HLA-DR-PE) was added to the cells. In addition, in two separate tubes, PE-conjugated mouse monoclonal CD44-PE antibody (Human MSC Analysis Kit, BD, Biosciences, San Jose, CA, USA) or PE-conjugated mouse monoclonal CD142-PE antibody (tissue factor, BD, Biosciences, San Jose, CA, USA) was added to hMSCs. After 20 min of incubation at 22 °C, cells were washed to remove excess antibodies. Analyses were carried out on a BD FACSCanto II or on a BD FACSCelesta using the BDFACS Diva software.

To detect the presence of positive markers for porcine MSCs, APC-conjugated mouse monoclonal CD90 antibody (Abcam, Cambridge, MA, USA), PE-conjugated mouse monoclonal CD105 antibody (Abcam, Cambridge, MA, USA), and APC-H7-conjugated mouse monoclonal CD29 antibody (BD Biosciences, San Jose, CA, USA) were used. The following antibodies were also used to detect the presence of negative markers: FITC-conjugated mouse monoclonal CD45 (Bio-Rad, Hercules, CA, USA), FITC-conjugated mouse monoclonal CD31 (Bio-Rad, Hercules, CA, USA), and FITC-conjugated mouse monoclonal HLA-DR (Thermo Fisher Scientific, Waltham, MA, USA). To reduce nonspecific binding, pMSCs at a cell concentration of 1 × 10^6^/ml were incubated with staining buffer containing 1% bovine serum albumin or 1% pig serum for 10 min. Antibodies were then added followed by a 15 min incubation period at 22 °C. Cells were washed and immediately analyzed by a BD FACSCanto II or a BD FACSCelesta using the BDFACS Diva software.

### Colony-forming unit fibroblast assay

The colony-forming unit fibroblast (CFU-F) assay was used as an indicator of progenitor cells as previously described [40]. Briefly, following the aforementioned culture durations and oxygen tensions, MSCs were plated at 100 and 200 cells per well on six-well plates in a total of 3 ml of CCM per well. Media were changed every 3–4 days and the cells were allowed to grow for 7–10 days. Prior to the overlap of colonies, cells were washed with PBS and fixed with chilled methanol for 10 min at room temperature. Next, the plates were allowed to air dry and stained with Giemsa to allow for visualization. Colonies larger than 50 cells were enumerated and reported as CFUs/well.

### Metabolic activity

MSCs were evaluated for their metabolic activity using the Vybrant assay (Thermo Fisher Scientific, Waltham, MA, USA), according to the manufacturer’s instructions. In this assay, non-fluorescent resazurin (R-12204) is reduced by viable cells to red-fluorescent resorufin during a 15 min incubation period. The reaction product exhibits absorption/emission at wavelengths of 563/587 nm, which was detected using a SpectraMax i3X system (Molecular Probes, Eugene, OR, USA). To perform this, MSCs were seeded at 1000 cells/cm in triplicates and their media evaluated along three different time points on days 3, 7, and 10 for the long-term culture or at 24 and 48 h for the short-term culture.

### Cell proliferation and viability

Following the metabolic assay, the MSCs were placed in a cell-lysis buffer (Cell Signaling Technology, Danvers, MA, USA). Following lysis at each time point, the multiwell plates were stored at − 80 °C until batch analysis. Next, plates were thawed and DNA concentration was measured using the Quant-iT PicoGreen assay (Invitrogen, Waltham, MA, USA) to evaluate cell proliferation, as previously described [40]. Briefly, an ultrasensitive fluorescent nucleic acid stain was used to quantify double-stranded DNA in solution. Samples were prepared by diluting with 1 × TE buffer (1:100) then plated in duplicates. Picogreen working solution is then added to prediluted samples. Plates were run on a SpectraMax i3X system (Molecular Probes, Eugene, OR, USA) and fluorescence measured at a wavelength of 502/523.

For qualitative assessment of proliferation in the long-term study, MSCs were stained with a fluorescent Live/Dead Cell Viability Kit, according to the manufacturer’s instruction (Life Technologies, Grand Island, NY, USA) and as previously described [41]. In this live/dead assay, the cytoplasm of viable cells is stained green (Ex/Em 495 nm/515 nm) and the nucleus of dead cells is stained orange (ex/em 528 nm/617 nm).

### Gene expression

To determine gene expression via quantitative real-time polymerase chain reaction (qRT-PCR), total RNA was extracted from MSCs using Trizol (Thermo Fisher Scientific, Waltham, MA, USA) and reverse-transcribed using a High-Capacity cDNA Archive Kit (Applied Biosystems, Foster City, CA, USA). The transcripts of interest were amplified from cDNA using Taqman Universal PCR Master Mix and all the primers were purchased from Applied Biosystems (Foster City, CA, USA). Amplification and detection were carried out with a StepOnePlus Real-Time PCR System (Applied Biosystems, Foster City, CA, USA) for high-mobility group box 1 (HMGB1), vascular endothelial growth factor (VEGF), tissue factor (F3), Nanog homeobox (NANOG), hypoxia inducible factor 1-alpha (HIF-1A), angiopoietin-1 (Ang-1), and the apoptotic genes cytochrome c (CYCS), B-cell lymphoma 2 (BCL2), bcl-2-like protein 4 (BAX), and caspase 3 (CASP3). Housekeeping genes used were 18 s for hMSCs and β-actin for pMSCs. Reference samples were (P2) MSCs prior to incubation in either hypoxia or normoxia cultures. All assays were done in duplicates and gene expression is expressed as a relative quotient (RQ) calculated from ΔΔCt of the sample of interest, where C_T_ is the threshold cycle.

$$ \Delta {C}_{T- Gene\ of\ Interest}={C}_{T- Housekeeping-}\ {C}_{T- Gene\ of\ Interest} $$

$$ \Delta \Delta {C}_{T- Gene\ of\ Interest}=\Delta {C}_{T- Gene\ of\ Interest}-\Delta {C}_{T- Reference} $$

$$ RQ={2}^{-\Delta \Delta {C}_{T- Gene\ of\ Interest}}. $$

### Secretion profile

The secretory profile of the pMSCs was assessed using the cytokine-chemokine 13-plex kit (Millipore, Billerica, MA, USA) to evaluate granulocyte-stimulating factor (GM-CSF), interferon gamma (IFN-γ), tumor necrosis factor alpha (TNF-α), and interleukin (IL) 1 alpha (IL-1α), IL-1β, IL-1ra, IL-2, IL-4, IL-6, IL-8, IL-10, IL-12, and IL-18. The same panel was used for hMSCs with the addition of fibroblast growth factor (FGF-2), platelet-derived growth factor (PDGF-AB/BB, and VEGF. To accomplish this, sample supernatant was spun down to remove any remaining cells and placed in − 80 °C until simultaneous analysis. Samples were run on a BioPlex 200 system (Bio-Rad, Hercules, CA, USA) following the manufacturer’s instructions. Data were standardized to the total amount of protein. For total protein, the Pierce™ 660 nm protein assay (Thermo Fisher Scientific, Waltham, MA, USA) was used according to manufacturer’s instructions and reported as picogram per gram of total protein (pg/gTP).

### Statistical analysis

Results are presented as means ± standard errors of the mean. All statistical tests were performed with the aid of GraphPad Prism version 7.01 (GraphPad Software, La Jolla, CA, USA). An unpaired, two-tailed Student’s *t* test was used for a two-group comparison. For the proliferation and metabolic assays in the long-term culture, a two-way analysis of variance (ANOVA) was used followed by a Tukey’s multiple comparisons post hoc test; a *p* value less than 0.05 was considered statistically significant.

## Results

### Hypoxia duration

In our first experiment, we set out to determine the optimal culture duration for MSCs under hypoxia. For these purposes, we tested culture times of 48 h, termed short term, and 10 days, termed long term, both under 1% and 21% oxygen tensions.

#### Surface marker expression

**Fig. 1:**
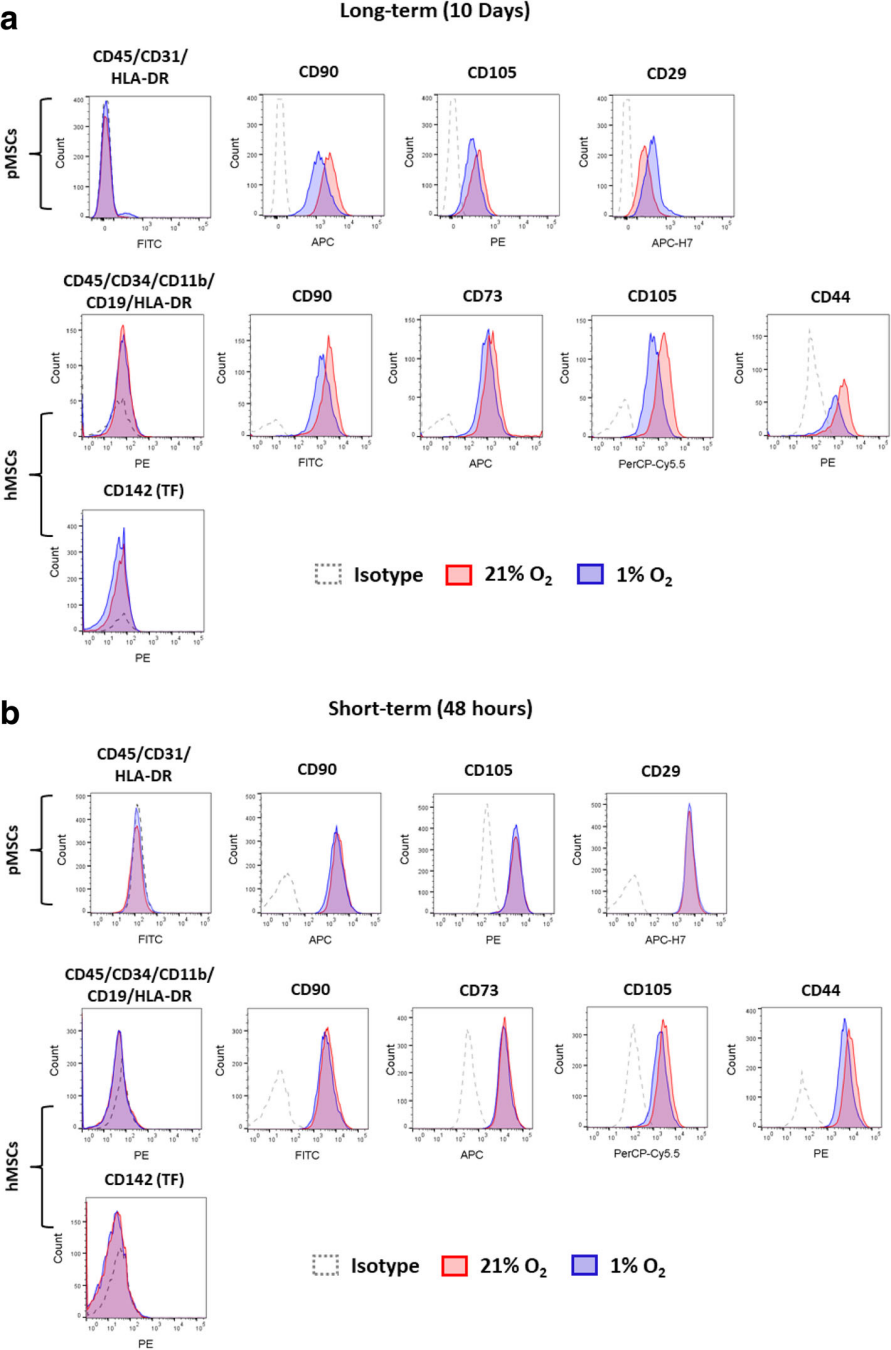
Surface expression of MSC markers under long-term and short-term hypoxia. (**a**) In long-term hypoxia, CD44 levels are decreased in hMSCs, especially under hypoxia. Levels of CD105 are decreased under hypoxia, in both human and porcine MSCs. No expression of TF (CD142) was evident in hMSCs under long-term normoxia or hypoxia culture. (**b**) In short-term hypoxia, all surface markers are nearly 100% besides CD105, which is decreased in hMSCs. No expression of TF was evident in hMSCs under short-term normoxia or hypoxia culture

Surface expression of common MSC markers in long-term and short-term hypoxia and normoxia cultures was evaluated using flow cytometry (Fig. 1). Under long-term hypoxia, the co-expression of negative markers was increased in pMSCs from 0.7% under normoxia to 2.8% under hypoxia. There were no changes in the co-expression of negative markers in hMSCs (normoxia 0.5%; hypoxia 0.1%). Interestingly, the percentage of cells co-expressing MSC markers was reduced in both species under long-term hypoxia. In hMSCs, 98.5% of cells co-expressed CD90, CD105, and CD73, which was reduced to 94.4%. Additionally, the expression of CD44 was reduced from 90% under normoxia to 75% under hypoxia. While there was no changes in the expression of CD90 (99.8–99.5%) and CD73 (99.3–98.9%), the expression of CD105 decreased from 99.4% under normoxia to 94.9% under hypoxia. The expression of tissue factor (TF) did not change under long-term hypoxia (normoxia 0.5%; hypoxia 0.1%). In pMSCs, the percentage of cells co-expressing CD90, CD105, and CD29 was reduced under long-term hypoxia. The percentage of these markers was also lower under long-term cultures compared to short-term cultures (in normoxia: from 98.2% under short-term to 85.5% under long-term; and in hypoxia: from 97.9% under short-term to 73.7% under long-term). The expression of CD90 did not change in long-term cultures (normoxia 99.8%; hypoxia 97.1%), whereas the expression of porcine CD105 was reduced from 91.7% under normoxia to 77.6% under hypoxia. The expression of CD29 was lower under long-term normoxia compared to long-term hypoxia (Fig. 1a).

Under short-term hypoxia, no differences were observed in the expression of negative markers of both hMSCs (normoxia 0.1%; hypoxia 0.0%) and pMSCs (normoxia 0.3%; hypoxia 0.8%). The percentage of hMSCs co-expressing CD90, CD105, and CD73 was reduced from 96.9% under normoxia to 90.7% under hypoxia, primarily due to reduction in the expression of CD105. Hypoxia did not affect the expression of CD44 (normoxia 99.8%; hypoxia 99.7%). Short-term hypoxia did not affect the expression of TF in hMSCs (normoxia 0.2%; hypoxia 0.5%). For pMSCs, co-expressions of MSC positive markers (CD90, CD105, and CD29) did not change under short-term hypoxia (Fig. 1b).

#### Cell metabolic, clonogenic, and proliferative capacities

**Fig. 2:**
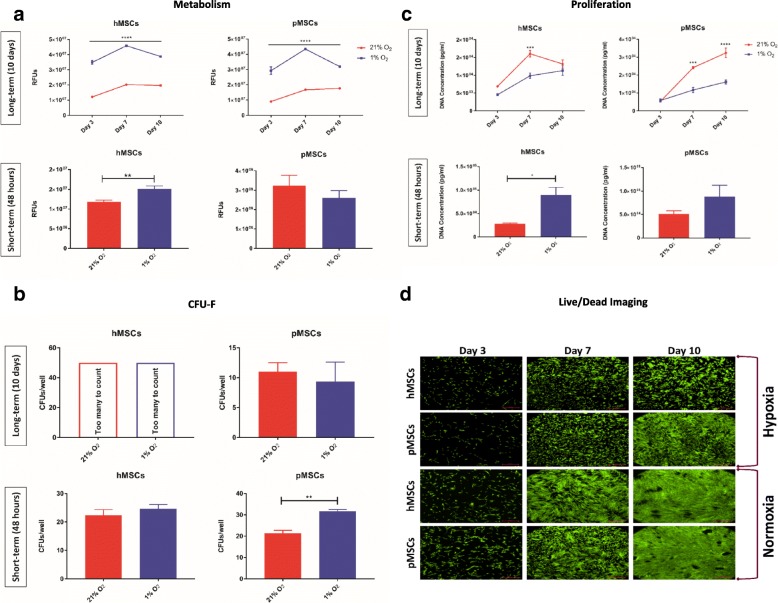
Characteristics of MSCs under long-term and short-term hypoxia. **a** Compared to normoxia, the metabolic activity of MSCs is significantly increased under 1% oxygen, particularly in long-term cultures; **b** In short-term culture, pMSCs show significant increase in self-renewing capacity as compared to normoxia (*p* < 0.01); **c** MSCs proliferate faster and generate greater cell yields under normoxia in long-term culture, while in short-term culture, MSCs demonstrate enhanced proliferation capacity under hypoxia; **d** In long-term culture, viable MSCs, shown in fluorescent green, reach a confluent state faster under normoxia; bar is 500 μm; ^*^p < 0.05; ^**^p < 0.01; ^***^p < 0.001; ^****^p < 0.0001

Throughout the study, the metabolic activity of both hMSCs and pMSCs was significantly higher in hypoxia in long-term culture, as compared to MSCs cultured in normoxia (*p* < 0.0001). In short-term culture the metabolic activity of hMSCs was significantly higher (*p* < 0.01) in hypoxia as compared to normoxia (Fig. 2a). In terms of clonogenicity, pMSCs cultured under short-term hypoxia exhibited significantly higher (*p* < 0.01) self-renewing capacity, as compared to those in normoxia (Fig. 2b). Cell proliferation was significantly higher (*p* < 0.001) at day 7 for both hMSC and pMSC in the long-term normoxia culture with significantly higher overall cell yields (*p* < 0.0001) in pMSCs cultured in normoxia. In comparison, in the short-term culture, hMSCs proliferated significantly faster (*p* < 0.05) in hypoxia than those cultured in normoxia (Fig. 2c). Qualitative data were in agreement. In the long-term study, fluorescent live/dead cell staining demonstrated that both pMSCs and hMSCs were more confluent in normoxia culture as compared to hypoxia (Fig. 2d).

#### Gene expression

**Fig. 3:**
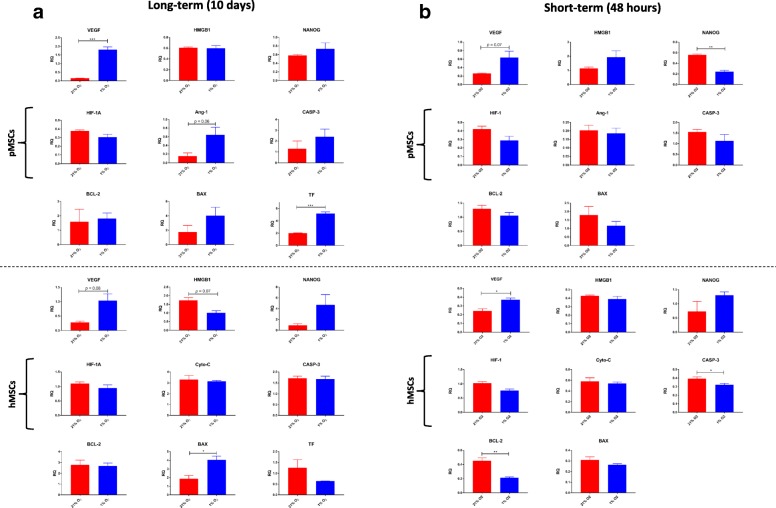
Gene expression of MSCs under long-term and short-term hypoxia. **a** In long-term cultures, TF, VEGF, and Ang-1 genes are upregulated in pMSCs under hypoxia. In hMSCs, VEGF (*p* = 0.08) and BAX (*p* < 0.05) were upregulated under hypoxia while HMGB1 (*p* = 0.08) was downregulated. (**b**) In short-term hypoxia culture, VEGF (*p* = 0.07) was upregulated while NANOG (*p* < 0.01) was downregulated in pMSCs. In hMSCs, VEGF (*p* < 0.05) was upregulated under hypoxia while the pro-apoptotic genes, BCL-2 (*p* < 0.01) and CASP-3 (*p* < 0.05), were downregulated. ^*^p < 0.05; ^**^p < 0.01; ^***^p < 0.001

Evaluation of gene expression revealed that in long-term cultures, TF (*p* < 0.001), VEGF (*p* < 0.001), and Ang-1 (*p* = 0.06) genes were upregulated in pMSCs under hypoxia. In hMSCs, VEGF (*p* = 0.08) and BAX (*p* < 0.05) were upregulated under hypoxia while HMGB1 (*p* = 0.08) was downregulated. In short-term hypoxia culture, VEGF (*p* = 0.07) was upregulated while NANOG (*p* < 0.01) was downregulated in pMSCs. In hMSCs, VEGF (*p* < 0.05) was upregulated under hypoxia while the pro-apoptotic genes, BCL-2 (*p* < 0.01) and CASP-3 (*p* < 0.05), were downregulated (Fig. 3).

#### Secretion profile

**Fig. 4:**
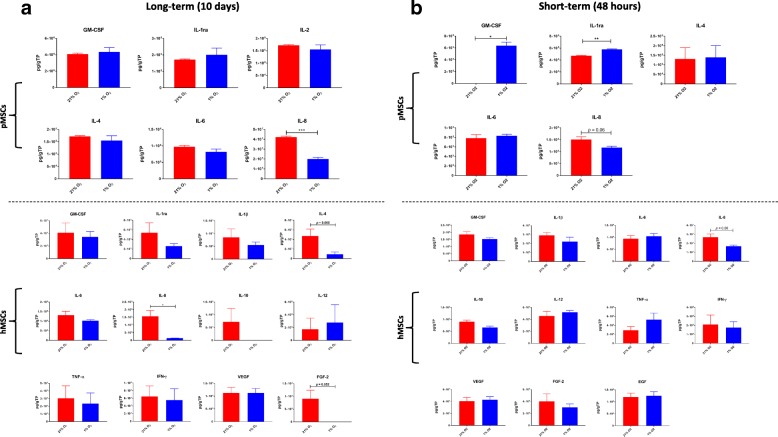
Secretion profile of MSCs under long-term and short-term hypoxia. (**a**) In long-term culture, IL-8 levels were suppressed under hypoxia in both human and porcine MSCs. (**b**) In short-term culture, IL-8 was similarly suppressed under hypoxia in both human and porcine MSCs (*p* < 0.06). In pMSCs, significantly higher levels of the anti-inflammatories, GM-CSF (*p* < 0.05) and IL-1ra (*p* < 0.01) were secreted under hypoxia, as compared to normoxia

The secretion profile of MSCs cultured under the different oxygen tensions was also assessed. In both long-term (*p* < 0.001 for pMSCs; *p* < 0.05 for hMSCs) and short-term (*p* = 0.06 for both) cultures, IL-8 levels were suppressed in hypoxia, as compared to normoxia. Additionally, in short-term culture, significantly higher levels of the anti-inflammatories, GM-CSF (*p* < 0.05) and IL-1ra (*p* < 0.01) were secreted from pMSCs cultured in hypoxia, as compared to levels in normoxia. Other bioactive factors that were tested and are not presented were below detection levels (Fig. 4).

### Hypoxia tension

Once we have determined that short-term hypoxia is optimal for MSC function, we wanted to both fine-tune the ideal culture time by testing both 24 and 48 h under hypoxia as well as evaluate two additional oxygen tensions, namely, 2% and 5% oxygen for MSC culture.

#### Surface marker expression

**Fig. 5:**
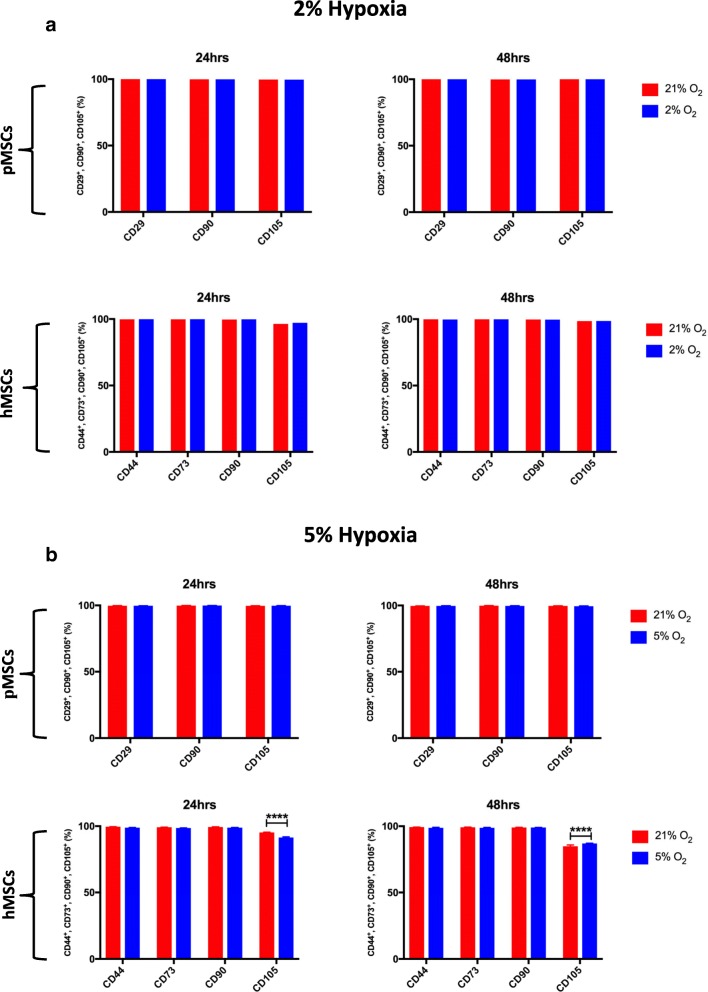
Surface expression of MSC markers under 2% and 5% short-term hypoxia. (**a**) Under 2% hypoxia, all MSC positive surface markers were above 95%. (**b**) In contrast, under 5% hypoxia, there was a significant reduction (*p* < 0.0001) in CD105 expression in hMSCs at noth 24 and 48 hours. This reduction was not evident in pMSCs where surface expression was maintained above 99%

Under both 2% and 5% hypoxia, expression of negative markers was extremely low in both human and porcine MSCs (< 1% and < 1.7%, respectively). Similarly, the expression of tissue factor was negligible in hMSCs under both 2% and 5% hypoxia (< 0.1% and < 0.4%, respectively). In the 2% study, all MSC positive surface markers were above 95%, irrespective of oxygen tension and time point. In contrast, under 5% hypoxia, there was a significant reduction (*p* < 0.0001) in CD105 expression in hMSCs, both in comparison to other surface markers as well as in response to hypoxia. This reduction was not evident in pMSCs, where surface expression was maintained above 99% (Fig. 5).

#### Cell metabolic, clonogenic, and proliferative capacities

**Fig. 6:**
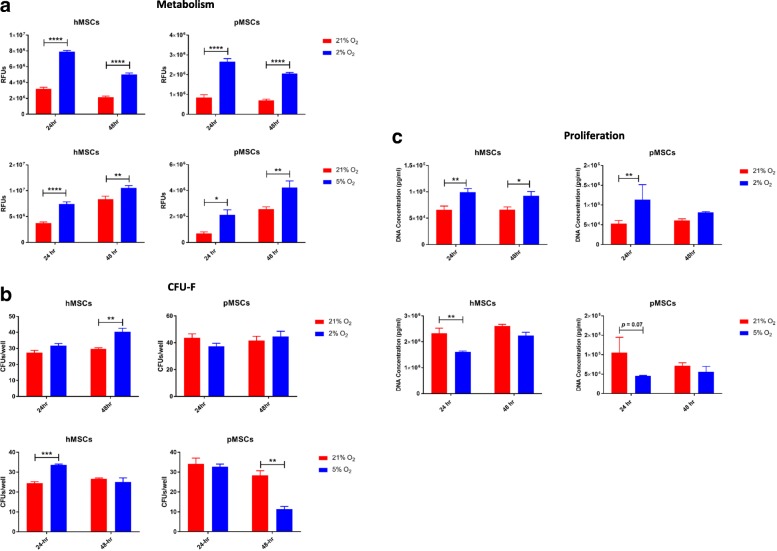
MSC characteristics under 2% and 5% short-term hypoxia. **a** The metabolic activity of MSCs was significantly elevated under both 2% and 5% hypoxia, when compared to normoxia; **b** The clonogenic capacity of hMSCs was significantly increased under hypoxia, while for pMSCs it was decreased after 48 h under 5% hypoxia; **c** Proliferation of MSCs was significantly increased under 2% hypoxia, whereas a significantly lower rate of proliferation was seen under 5% hypoxia; ^*^p < 0.05; ^**^p < 0.01; ^***^p < 0.001; ^****^p < 0.0001

Similar to 1% hypoxia, the metabolic activity of MSCs was significantly elevated in both 2% (*p* < 0.0001 for both species and durations) and 5% (in hMSCs, *p* < 0.0001; in pMSCs, *p* < 0.05 at 24 h; *p* < 0.01 for both species at 48 h) hypoxia, when compared to normoxia (Fig. 6a). Similarly, the clonogenic capacity of hMSCs was significantly increased under hypoxia (*p* < 0.01 in 2% at 48 h and *p* < 0.001 in 5% at 24 h), while for pMSCs it was significantly decreased after 48 h under 5% hypoxia (Fig. 6b). Similar to short-term culture in 1% hypoxia, proliferation of MSCs was significantly increased under 2% hypoxia (*p* < 0.01 for both species at 24 h; and in hMSCs, *p* < 0.05 at 48 h), whereas a significantly lower rate of proliferation (in hMSCs, *p* < 0.01 at 24 h and in pMSCs *p* = 0.07 at 24 h) was seen under 5% hypoxia (Fig. 6c).

#### Gene expression

**Fig. 7:**
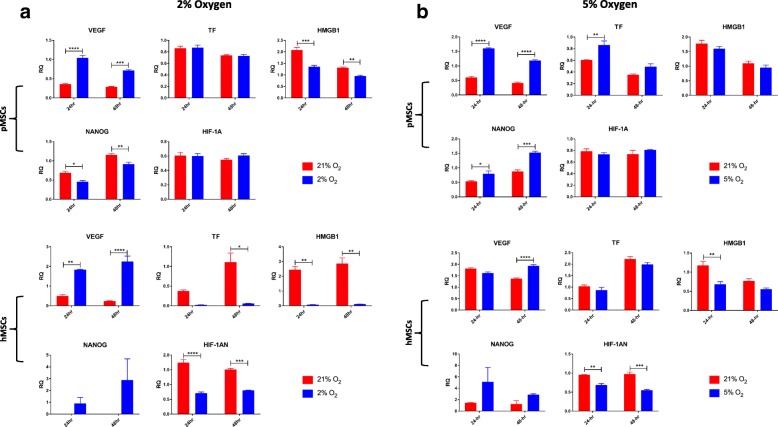
Gene expression under 2% and 5% short-term hypoxia. (**a**) Under 2% short-term hypoxia, there was a significant upregulation in the angiogenic gene, VEGF, with a concomitant downregulation in the expression of HMBG1 in both human and porcine MSCs. Additionally, under hypoxia, the stem cell gene, NANOG, was downregulated in porcine, but not human MSCs, while TF and HIF antagonist were downregulated in human, but not porcine MSCs. (**b**) Similar to 2% hypoxia, under 5% hypoxia, the VEGF gene was significantly upregulated under hypoxia in both human and porcine MSCs, while HMGB1 and HIF antagonist were downregulated in hMSCs. As opposed to 2% hypoxia, TF was significantly upregulated in pMSCs under 5% hypoxia; ^*^p < 0.05; ^**^p < 0.01; ^***^p < 0.001; ^****^p < 0.0001

Similar to 1% hypoxia, there was a significant upregulation in the angiogenic gene, VEGF, in MSCs cultured under both 2% (in pMSCs, *p* < 0.0001 at 24 h and *p* < 0.001 at 48 h; in hMSCs, *p* < 0.01 at 24 h and *p* < 0.0001 at 48 h) and 5% hypoxia (in pMSCs, *p* < 0.0001 at 24 and 48 h; in hMSCs, *p* < 0.0001 at 48 h). Conversely, there was a significant downregulation in the expression of HMBG1 in both 2% (in pMSCs, *p* < 0.001 at 24 h and *p* < 0.01 at 48 h; *p* < 0.01 for hMSCs at 24 and 48 h) and 5% (in hMSCs, *p* < 0.01 at 24 h) hypoxia. The stem cell gene, NANOG, was downregulated under 2% hypoxia (in pMSCs, *p* < 0.05 at 24 h and *p* < 0.01 at 48 h), while under 5% hypoxia, it was significantly upregulated (in pMSCs, *p* < 0.05 at 24 h and *p* < 0.001at 48 h). Finally, under 2% hypoxia, the thrombogenic gene, TF, was significantly downregulated (*p* < 0.05) in hMSCs; whereas under 5% hypoxia it was significantly upregulated (*p* < 0.01 at 24 h) in pMSCs only (Fig. 7).

#### Secretion profile

**Fig. 8:**
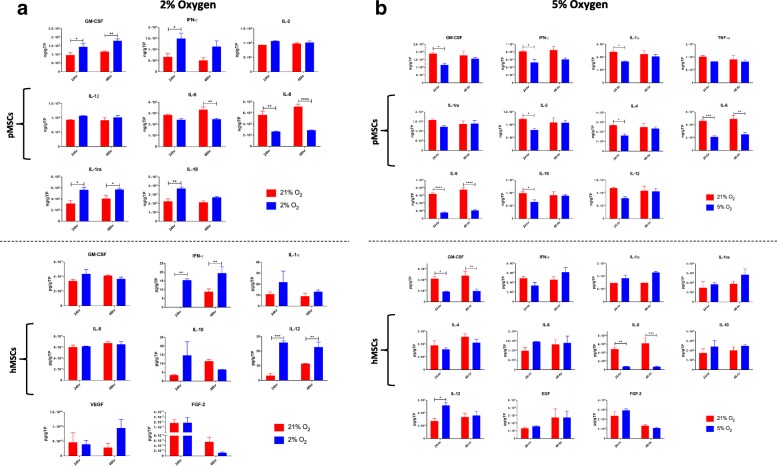
Secretion profile of MSCs under 2% and 5% short-term hypoxia. (**a**) Under 2% short-term hypoxia, IL-6 and IL-8 secreted by pMSCs were significantly suppressed while GM-CSF, IFN-γ, IL-1ra, and IL-18 were significantly increased. In hMSCs, IFN-γ and IL-18 were significantly increased under 2% hypoxia. (**b**) Similar to 2% hypoxia, 5% hypoxia significantly suppressed the secretion of the pro-inflammatories IL-6 and IL-8 in pMSCs. Unlike 2%, 5% hypoxia concomitantly suppressed the secretion of GM-CSF, IFN-γ, IL-1α, IL-2, IL-4, and IL-10. In hMSCs, as in pMSCs, IL-8 and GM-CSF levels were suppressed, while the secretion of IL-12 was increased under hypoxia, similar to the 2% hypoxia; ^*^p < 0.05; ^**^p < 0.01; ^***^p < 0.001; ^****^p < 0.0001

As in 1% hypoxia, levels of secreted IL-8 were significantly suppressed under both 2% (in pMSCs, *p* < 0.01 at 24 h and *p* < 0.001 at 48 h) and 5% (in pMSCs, *p* < 0.0001 at 24 and 48 h; in hMSCs, *p* < 0.01 at 24 h and *p* < 0.001 at 48 h) hypoxia cultures. Similar to 1%, GM-CSF was increased in pMSCs under 2% hypoxia (*p* < 0.05 at 24 h and *p* < 0.01 at 48 h), while under 5% hypoxia it was significantly decreased (in pMSCs, *p* < 0.05 at 24 h; in hMSCs, *p* < 0.05 at 24 h and *p* < 0.01 at 48 h). Likewise, as in 1% hypoxia, levels of IL-1ra were significantly increased in pMSCs under 2% hypoxia (*p* < 0.05 at 24 and 48 h), but no change was detected under 5% hypoxia. Additionally, under 2% hypoxia, there was a significant increase in secretion of IFN-γ (in pMSCs, *p* < 0.05 at 24 h; in hMSCs, *p* < 0.01 at 24 and 48 h) while the opposite effect was seen under 5% hypoxia (in pMSCs, *p* < 0.05 at 24 h). IL-12 levels were significantly higher only in hMSCs cultured under 2% (*p* < 0.001 at 24 h and *p* < 0.01 at 48 h) and 5% hypoxia (*p* < 0.05 at 24 h). IL-18 was also significantly increased in 2% hypoxia, but only in pMSCs, (*p* < 0.01 at 24 h). Under 5% hypoxia, there was a significant decrease in the levels of IL-1a, IL-2, IL-4, and IL-10 (*p* < 0.05 at 24 h) in pMSCs only. Finally, similar to IL-8 suppression, levels of IL-6 were significantly diminished in both 2% (*p* < 0.01 at 48 h) and 5% hypoxia (*p* < 0.001 at 24 h and *p* < 0.01 at 48 h), albeit in pMSCs only (Fig. 8).

## Discussion

Emerging evidence indicates that hypoxia culture preserves the stemness of MSCs. However, key questions, such as the optimal culture time and oxygen tension, still remain unanswered with some conflicting reports. Kim et al. reported that approximately 2 weeks in hypoxia showed increased MSC proliferation and viability as compared to normoxia [11]. However, they did not report whether a dedicated environmental chamber was used in their study, rather than an oxygen-controlled cell-culture incubator, which interferes with the hypoxia state by permitting the transient exposure of cells to ambient oxygen during routine culture maintenance. Saraswati et al. reported that a 72 h exposure (which they defined as prolonged) induced monocarboxylate transporter 4 (MCT4) expression, which led to increased lactate and cell apoptosis within the wound bed [42]. Pattappa et al. demonstrated that continuous hypoxia reduced clonogenic abilities and impaired osteogenic differentiation potential of MSCs [43]. Other studies showed that short-term hypoxia is beneficial for MSC function for various indications [44–46].

In this study, we demonstrate that both long-term and short-term hypoxia can augment the functional characteristics of MSCs, as compared to standard cultures (i.e., normoxia); yet, MSCs in short-term hypoxia exhibited superior functional characteristics than those cultured under long-term hypoxia. In a second set of experiments, we further evaluated different oxygen tensions (i.e., 1%, 2%, and 5%) to determine the optimal pre-conditioning conditions under hypoxia. We observed that MSCs exhibit superior functionality under all hypoxia conditions, as compared to normoxia, but preconditioning MSCs for 48 h under 2% appears to promote the most favorable outcome.

In long-term hypoxia, surface expression of common MSC markers dramatically dropped for both human and porcine MSCs. This was most notable with reduction in CD105 and CD44 markers. Similarly, Roemeling-van Rhijn and colleagues reported a reduction in CD105 in adipose-derived MSCs after 10 days in 1% hypoxia [35]. In short-term hypoxia, a reduction in CD105 only occurred in hMSCs under 1% hypoxia with no concomitant reduction in CD44, while no change was noted in 2% and 5% hypoxia. We also evaluated the expression of TF (CD142) on hMSCs since some studies have reported an increase in TF expression during culture [47–49]. Importantly, no TF expression was evident in our human bone marrow MSCs either in short-term or long-term cultures under hypoxia or normoxia. It is important to note, however, that depending on their passage number (i.e., population doublings), adipose-derived MSCs can inherently express TF since they are derived from the stromal vascular fraction. In our experience, this TF expression is reduced as the cells become more homogeneous and MSC-like with increasing passages (data not shown). Similarly, as reported by Gleeson et al. [49] and from our experience, pMSCs tend to be more thrombogenic than hMSCs, which was evident in this study through an upregulation in TF after long-term hypoxia.

The metabolic activity of the MSCs was significantly increased under all hypoxia conditions (Figs. 2a, 6a). These findings are similar to those reported in other studies and are most likely attributed to a shift from aerobic respiration via oxidative phosphorylation to anaerobic respiration via glycolysis [50–52]. Conversely, the proliferation rate of MSCs was significantly higher under normoxia in the long-term (10-day) culture (Fig. 2c, d). This could be because the MSCs have shifted their biological function from proliferation to lower energy-consuming processes in order to adapt to the ‘chronic’ low oxygen state. This is in comparison to the short-term hypoxia in which the cells are transiently (up to 48 h) exposed to hypoxia and therefore may have not undergone this switch in function. The MSCs in the short-term hypoxia culture had increased proliferation capacity under all oxygen tensions, as compared to those cultured in normoxia (Figs. 2c-d and 6c). The clonogenic capacity of the cells was in agreement with their proliferative function; that is, the ability of MSCs to self-renew was significantly increased in short-term hypoxia under all oxygen tensions (Figs. 2b and 6b). These data agree are in agreement with the study by Boyette et al., who reported enhanced proliferation and clonogenic potentials following culture in 5% hypoxia [38].

We also evaluated gene expression levels under hypoxia, as compared to normoxia. Upregulation of the VEGF gene was evident in all hypoxia cultures, which is consistent with other studies [53–56]. Such upregulation may have important clinical implications since VEGF and Ang-1 have been shown to play pivotal roles in angiogenesis, alveolar development, and treatment of acute lung injury (ALI) [8, 57–60]. The concomitant upregulation in the angiogenic protein, Ang-1, further reinforces this point. The significant downregulation in HMGB1 under all hypoxia cultures further demonstrate the favorable effect of hypoxia on MSC function. HMGB1 is a protein that is expressed by various immune cells and MSCs in response to tissue damage. Therefore, its downregulation in hypoxia may signify cytoprotection from cellular damage, such as production of reactive oxygen species, as seen in normoxia cultures [61, 62]. In terms of apoptosis, the BAX gene was significantly upregulated in hMSCs under long-term hypoxia (*p* < 0.05). The BAX gene primarily functions as an apoptotic activator by destabilizing the mitochondrial channel triggering the release of cytochrome C; however, other apoptotic genes (cyto-c and CASP3) were not upregulated. In contrast, in short-term culture, hypoxia exposure inhibited the apoptotic pathway demonstrated via the downregulation of the apoptotic genes, BCL-2 (*p* < 0.01) and CASP3 (*p* < 0.05) in hMSCs, as compared to normoxia. The same trends were seen with the pMSCs, though differences were not significant. Finally, extended hypoxia triggered a significant upregulation (*p* < 0.001) in TF in pMSCs, but not hMSC, which corroborated the flow cytometry results (i.e., no surface expression of TF in hMSCs). Upregulation of TF indicates a thrombogenic disposition of these pMSCs. The fact that TF was not upregulated in hMSCs demonstrates inter-species differences in MSCs, an important fact for their translation to the clinic.

Recently, emergent evidence indicates that MSCs impart their therapeutic function predominantly via paracrine secretion of bioactive factors. Therefore, we evaluated the secretion profile from MSCs cultured under hypoxia, as compared to normoxia. Most notably, IL-8 levels were significantly suppressed under all hypoxia conditions. The IL-8 chemokine, produced by various immune cells and MSCs, is a potent neutrophil attractant and activator [63]. Neutrophils play a central role in the progression of ALI to acute respiratory distress syndrome (ARDS). Moreover, IL-8 has been shown to be present at high concentrations in bronchoalveolar lavage fluids of ARDS patients [64, 65], which was also associated with increased mortality [66, 67]. Therefore, in regards to MSC therapy for ARDS, mitigating the secretion of IL-8 by pre-conditioning them with hypoxia prior to administration may potentially offer benefit at the bedside. In addition to IL-8, significantly higher levels of the anti-inflammatories, GM-CSF and IL-1ra were secreted from MSCs cultured in short-term 1% and 2%, but not 5% hypoxia. Both IL-1ra and GM-CSF have been shown to play instrumental roles in the resolution of ARDS [64, 68–71]. As with IL-8, IL-6 secretion was also suppressed, but only in pMSCs under 2% and 5% hypoxia. IL-6 is a potent pro-inflammatory cytokine that plays a key role in the pathogenesis of various inflammatory disorders, such as ARDS. Increased levels of both IL-8 and IL-6 have been shown to be highly correlated with the incidence of ARDS and overall mortality [72–74]. The concomitant increase in IFN-γ following IL-12 secretion by hMSCs under hypoxia was expected since IL-12 promotes the production of IFN-γ. Similarly, under 2% hypoxia, the secretion of the pro-inflammatory IL-18, which works in concert with IL-12 to promote cell-mediated immunity, was also significantly enhanced, but in pMSCs only. As with TF expression, secretion of different bioactive factors by MSCs of different species demonstrates inter-species variation in MSC characteristics and function.

## Conclusions

In this study, we demonstrate that hypoxia augments the therapeutic characteristics of both porcine and human MSCs. Overall, short-term exposure to hypoxia is superior to long-term culture. This is mainly exemplified by the increase in proliferation, self-renewing capacity, regulation of key genes, and modulation of the inflammatory milieu. These data highlight the importance of down-selecting the appropriate oxygen tension for generating robust MSCs for clinical applications. Undoubtedly, augmenting the anti-inflammatory properties of MSCs through short-term hypoxia pre-conditioning will increase their efficacy for treating a large array of conditions. Our results will be corroborated in vivo in a preclinical model of ARDS manifested, in part, via systemic hypoxemia and acute inflammation.

## Abbreviations

ALI: Acute lung injury
AML: Acute myeloid leukemia
Ang-1: Angiopoietin 1
ANOVA: Analysis of variance
ARDS: Acute respiratory distress syndrome
CCM: Complete culture media
CFU-F: Colony-forming unit fibroblast
Ct: Threshold cycle
GM-CSF: Granulocyte-macrophage colony stimulating factor
HMGB1: High-mobility group box 1
IFN-γ: Interferon gamma
IL: Interleukin
MNCs: Peripheral blood mononuclear cells
MSCs: Mesenchymal stem cells
qRT-PCR: Quantitative real-time polymerase chain reaction
TF: Tissue factor
TNF-α: Tumor necrosis factor alpha
VEGF: Vascular endothelial growth factor
